# Novel Insights into the Genetic Landscape of Nonalcoholic Fatty Liver Disease

**DOI:** 10.3390/ijerph16152755

**Published:** 2019-08-01

**Authors:** Alice Emma Taliento, Marcello Dallio, Alessandro Federico, Daniele Prati, Luca Valenti

**Affiliations:** 1 Translational Medicine, Department of Transfusion Medicine and Hematology, Fondazione IRCCS Ca’ Granda Ospedale Maggiore Policlinico IRCCS, 20122 Milan, Italy; 2 Department of Precision Medicine, University of Campania “Luigi Vanvitelli”, 80131 Naples, Italy; 3 Department of Pathophysiology and Transplantation, University of Milan, 20122 Milan, Italy

**Keywords:** liver, nonalcoholic fatty liver disease, hepatocellular carcinoma, steatosis, genetics, retinol, oxidative stress

## Abstract

Nonalcoholic fatty liver disease (NAFLD), the most common liver disorder worldwide, is epidemiologically associated with overweight, insulin resistance features and type 2 diabetes, and can progress to advanced liver fibrosis and hepatocellular carcinoma. Genetic factors play an important role in the development of NAFLD, which is a multifactorial disease. Several common naturally occurring variants modulating lipid and retinol metabolism in hepatocytes predispose to NAFLD development and progression, in particular those in *PNPLA3*, *TM6SF2*, *MBOAT7*, and *HSD17B13*. In addition, genetic variants that protect hepatic cells from oxidative stress modulate the susceptibility to progressive NAFLD. Although the molecular mechanisms linking these genetic variants with liver disease are not yet fully understood, hepatic fat has emerged as a major driver of the disease, while altered retinol metabolism and mitochondrial oxidative stress play a role in determining the development of advanced NAFLD.

## 1. Nonalcoholic Fatty Liver Disease

Nonalcoholic fatty liver disease (NAFLD) is defined as excessive fat accumulation in hepatocytes (exceeding 5% of liver weight), which is not caused by at risk alcohol consumption (>20/30 g day in females/males). NAFLD has already became the most common liver disease worldwide [1] and its prevalence is still increasing [2]. NAFLD has a strong epidemiological association with overweight, insulin resistance features and metabolic syndrome, in particular type 2 diabetes (T2D) [3]. In this pathological condition, neutral lipids are accumulated in the lipid droplets within hepatocytes. The neutral fat is mainly stored in the form of triglycerides throughout esterification, which is caused by hyperinsulinemia-driven de novo lipogenesis, increased influx of free fatty acids coming from the insulin-resistant and inflamed adipose tissue, and intestinal absorption from the diet. The esterification process is a protective mechanism to safely store fatty acids, which would otherwise activate lipotoxicity, leading to fibrogenesis and liver disease [4]. Several proteins are involved in the regulation of lipid droplets metabolism, which is highly complex [5].

NAFLD is a heterogeneous condition, as it encompasses a broad clinical spectrum of liver damage, ranging from pure steatosis to nonalcoholic steatohepatitis (NASH). NASH is a chronic liver disease defined as the presence of steatosis, together with both lobular necro-inflammation and hepatocellular damage (“ballooning”) [6]. NASH can frequently lead to hepatic fibrosis that may evolve to advanced liver diseases, such as cirrhosis, and finally hepatocellular carcinoma (HCC). NAFLD and the subsequent possible transition to liver fibrosis are multifactorial processes, meaning that both genetic factors and environmental factors, such as microbiota composition and gut permeability, proinflammatory imbalance, and oxidative stress, are involved [7]. This review will delineate the most recent advancements in the genetics of NAFLD, and the novel therapeutic implications of these progresses [8,9,10].

## 2. The Overall Role on Inherited Factors

NAFLD is a multifactorial disease, but genetic factors influencing hepatic fat accumulation strongly impact on the risk of disease development. This concept was demonstrated by multiple approaches [8]. First, twin studies performed in the general adult population have shown that in individuals without viral hepatitis or alcohol abuse, more than half of the hereditability of aminotransferases levels, which were strongly associated with hepatic fat content, is genetically determined [11]. A strong heritability of hepatic fat content and liver damage has recently been confirmed with the evaluation of liver fat and fibrosis by means of nuclear magnetic spectroscopy and elastography in a population-based twin study [11,12]. Indeed, genetic effects explained 55% of the variation of aminotransferases levels, while the contribution of environmental factors explained the remaining risk [11]. Furthermore, steatosis and fibrosis heritable components were shared in the cohort, together with metabolic comorbidities, suggesting that dysmetabolism and hepatic fat accumulation play a causal role in triggering progressive liver disease.

Second, population-based studies conducted in multi-ethnic cohorts have shown that the susceptibility towards NAFLD has a strong inter-ethnic variability. Indeed, African-Americans have a reduced tendency to develop NAFLD as compared to Europeans, while Asians and particularly Hispanics, are at increased risk [13]. The inter-ethnic differences were not accounted for by T2D, adiposity, and socioeconomic factors.

Finally, family studies have shown that there is a tendency for severe cases of NAFLD to cluster in specific pedigrees, as demonstrated by the several-fold higher risk of NAFLD progressing to advanced liver fibrosis (prevalence 18%) in first-degree family member of patients with NASH cirrhosis, as compared to the general population (1%) [14]. In accordance with these results, individuals with a family history of NAFLD displayed a higher risk of developing the disease, in particular when both parents were affected, or in individuals without cardiovascular risk factors [15].

## 3. PNPLA3 and HSD17B13: The Role of Lipid Droplets and Retinol Metabolism

During the last 10 years, genome wide association studies have allowed the uncovering of the main common genetic determinants of NAFLD and hepatic fat content. The rs738409 C>G single nucleotide polymorphism (SNP) that encodes for the I148M protein variant of *Patatin-like phospholipase domain-containing 3* (*PNPLA3*) is the one with the greatest impact (Table 1). Previously, a genome-wide association study (GWAS) performed in a large population comprising different ethnicities has shown that the I148M variant is most common in Hispanics, the group most susceptible to NAFLD [16].

The I148M variant has been linked to higher hepatic fat content, without a major direct effect on insulin resistance and adiposity features [16]. The I148M variant determines an increase in the risk of the full spectrum of clinical manifestations associated with NAFLD, from simple steatosis to inflammation and NASH, and the subsequent transition towards fibrosis and cirrhosis [9,24,25]. Therefore, this protein is now considered as a major player in liver disease progression. Recently, studies in European populations have demonstrated that individuals who develop HCC have a nine-fold enrichment I148M variant in the prevalence of homozygosity for the I148M variant, as compared to the general population. On the other hand, the absence of the variant could rule out the risk of developing HCC related to NAFLD with a high specificity [9].

Lifestyle features, such as the intake of fructose-enriched drinks and lack of physical activity, interact with the I148M variant in triggering hepatic fat accumulation and NAFLD development, particularly in children [24,26]. However, excessive adiposity is the major environmental determinant of the phenotypic expression of the I148M *PNPLA3* variant, as concerning the development of liver disease, in individuals who do not exceed in alcohol consumption [27]. Therefore, environmental factors, such as adiposity, contribute to NAFLD progression by interacting with genetic risk variants. The evidence of an epidemiological interaction between adiposity and genetic variants in determining NAFLD predisposition is supported for *PNPLA3* by functional data. In obesity and insulin resistance conditions, *PNPLA3* expression is induced by insulin and hepatocytes and hepatic stellate cells, and localizes at the surface of lipid droplets [28]. Indeed, the *PNPLA3* promoter carries a specific consensus site for the Sterol regulatory element-binding protein 1c (SREBP-1c) that is induced by insulin [29,30]. Liver X receptor (LXR) mediates SREBP-1c upregulation, and *PNPLA3* mRNA levels increase after LXR agonist treatment [31,32]. In the presence of hyperinsulinemia associated with excess adiposity, the mutated I148M PNPLA3 protein accumulates at the surface of lipid droplets, where it is not catalytically active, and evades ubiquitylation, altering triglycerides and phospho-lipids turnover and remodeling [33,34]. The mechanism seems at least partially mediated by the ability of PNPLA3 variant to sequester CGI-58, which is an essential cofactor for the activity of ATGL/PNPLA2, the main triglycerides hydrolase of hepatocellular lipid droplets [35].

Furthermore, the PNPLA3 protein plays a role in the release of retinyl-palmitate, the storage form of retinol, whose metabolites (retinoids, that is vitamin A) are involved in the regulation of gene transcription by binding nuclear hormone receptors (RAR/RXR). In hepatic stellate cells (HSC), the main cellular site of retinol storage in the human body and a key player in the liver fibrogenic process, impaired *PNPLA3* expression causes a reduced dismissal of retinol. The I148M variant induces the decrease of the PNPLA3 retinyl-palmitate lipase activity leading to higher fat content in HSC droplets, and is associated with a proinflammatory and profibrogenic phenotype [36]. In addition to steatosis, alteration in the release of retinol by HSC may therefore lead to hepatic inflammation, fibrogenesis, and carcinogenesis.

In keeping with a major role played by retinol in the pathogenesis of progressive NAFLD, a recent study in large independent cohorts of individuals from the general population demonstrated that a single nucleotide polymorphism in *Hydroxysteroid 17-beta dehydrogenase 13* (*HSD17B13*, rs72613567:TA), encoding for a splice variant, was associated with protection against both alcoholic and nonalcoholic progressive fatty liver disease at exome-wide level [37] (Table 1). The protein is expressed at the surface of hepatocellular lipid droplets and the variant generates a truncated unstable protein and causes a reduction in the HSD17B13 enzymatic activity [37]. Interestingly, the impact of the variant on the protection against NAFLD was larger in carriers of the *PNPLA3* I148M variant, suggesting the two risk factors interact in the pathogenesis of this condition. A major role of *HSD17B13* in NAFLD predisposition has already been confirmed. Indeed, in a case-control study in 429 patients with histological NAFLD and 180 controls from South America, the minor rs72613567 allele protected against NASH and fibrosis [17]. The spliced variant was linked with decreased HSD17B13 levels in hepatocytes [17].

Recently, another variant in *HSD17B13* (rs62305723, encoding for the p.P260S aminoacidic substitution) was associated with reduced inflammation and ballooning in a large cohort of patients with histological NAFLD [38]. A key finding was that the HSD17B13 protein has retinol dehydrogenase (RDH) activity, depending on the availability of cofactors and on the correct targeting to lipid droplets [39]. Both rs72613567 splice variant and rs62305723 p.P260S variant resulted in loss of enzymatic activity and protection against liver damage associated with NAFLD [38].

**Figure 1:**
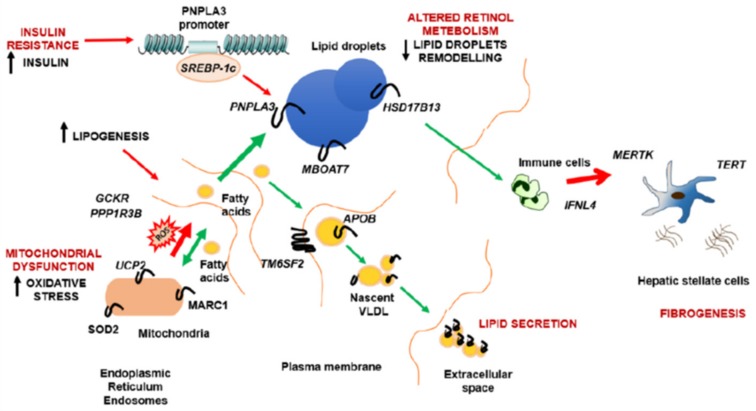
The possible molecular mechanisms beyond the genetic variants that are associated to liver damage in NAFLD are shown.

As both the PNPLA3 and HSD17B13 proteins are involved in retinol metabolism, these data suggest that genetic variation affecting retinol metabolism at the level of HSC or hepatocytes lipid droplets may have a role in the pathogenesis of progressive NAFLD [39]. Therefore, the remodeling of specific lipids and retinol has an important function in the development of NAFLD, contributing to fat accumulation, inflammation, and fibrogenesis. The possible molecular mechanisms beyond the genetic variants that are associated to liver damage in NAFLD are shown in Figure 1.

## 4. Impact of Genetic Variation in Nuclear-Encoded Mitochondrial Protein

During the development of NAFLD, oxidative stress plays an important role in triggering the hepatic damage. Insulin resistance is characterized by an overload of free fatty acids in the mitochondria, that can induce reactive oxygen species (ROS) overproduction and an increase the permeability of the inner mitochondrial membrane. This process leads to mitochondrial dysfunction and may trigger apoptosis [40]. In addition to free fatty acids overload, previous studies on large cohorts of NAFLD patients showed that accumulation of iron stores catalyzes an increase of oxidative stress causing fibrosis and inflammation [41,42].

Contemporary studies have identified candidate genes that are involved in NAFLD pathogenesis and play a role in the oxidative stress regulation. First, a family study evaluating children with fibrotic NAFLD and their parents demonstrated a significant association between a common polymorphism (C47T, rs4880 variant) in the *SOD2* gene, encoding for the manganese-dependent superoxide dismutase (MnSOD), and more advanced fibrosis [43]. In keeping with these results, another study in a Japanese cohort of patients with NASH showed that the C47T *SOD2* variant was more represented in patients with NASH [44]. MnSOD is a mitochondrial matrix enzyme that protects cells from toxic radicals, facilitating the conversion of superoxide into hydrogen peroxide and di-atomic oxygen, and compensating for increased generation of reactive oxygen species [45]. The C47T variant generates an amino acid substitution in the signal sequence, resulting in an alteration of the efficiency of import into the mitochondrial matrix [46]. Although the specific molecular mechanism underlying the effect of this variant and its correlation to NAFLD phenotype are not yet understood, a recent animal study showed that when NASH was induced in mice by a high-fat diet, SOD2 activity was induced [47].

Furthermore, a non-coding variant in the promoter (-55C>T, rs1800849) of *UCP3*, encoding for the uncoupling protein 3, a mitochondrial transporter that increases the proton leak of the inner membrane of mitochondria and uncouples the oxidative phosphorylation, has been shown to be involved in NAFLD susceptibility [48]. The rs1800849 variant was reported to increase *UCP3* mRNA expression levels in the skeletal muscle [49], especially in overweight individuals [49], and to predispose to an atherogenic lipid profile [50]. A recent study in a cohort of overweight patients with NAFLD reported an association between the -55C>T variant and insulin resistance, severe steatosis and inflammation [51].

In addition, increased hepatic expression of Uncoupling protein 2 (UCP2), which is involved in the regulation of mitochondrial lipid efflux and oxidative metabolism [52], has been detected in NASH patients as compared to controls. The -866 G>A variant (rs695366), located at the promoter region of *UCP2*, has been linked to higher gene expression and insulin sensitivity. In keeping, in a multicenter cohort of patients at risk of NASH, it has been reported that homozygosity for the 866A *UCP2* variant is associated with protection against liver damage related to NAFLD, especially in patients without T2D [52].

Very recently, Connor et al. identified and preliminarily reported a rare missense variant in *MARC1* (A165T), encoding for the Mitochondrial Amidoxime Reducing Component 1, as associated with protection against all causes of cirrhosis in the general population [53]. This variant results in a truncated protein, likely determining a loss-of-function, and has been associated with lower levels of hepatic fat. Furthermore, *MARC1* p.A165T has been associated with reduced levels of several biomarkers of liver disease. The A165T variant occurs at the N-terminal domain of *MARC1* that anchors the protein to the outer membrane of the mitochondria. Although the molecular mechanism of MARC1 enzymatic activity has not yet been understood, previous work has shown that the protein is involved in the neutralization of ROS during oxidative stress by reducing nitrite to produce nitric oxide [54] and detoxifying trimethylamine N-oxide [55]. Therefore, further investigations may provide insight into the specific role of *MARC1* in oxidative stress regulation in mitochondria and on the association with hepatic inflammation.

## 5. Impact of Genetic Variation Influencing Inflammation and Fibrogenesis

After steatosis development, genetic variants that modulate downstream pathways activate inflammation and fibrosis, thereby modulating the progression of liver damage in susceptible individuals with NAFLD. Induction of innate immunity pathways constitute one of the main players in NASH. Variation in the *Interleukin 28* (*IL28*) locus encodes for the alternative Interferon lambda-3 and lambda-4 proteins. The rs368234815 δG>TT variant is associated with the expression of genes stimulated by interferons and with protection against severe inflammation and development of advanced fibrosis in patients with NAFLD [18,56].

Moreover, variation at the *Mer T kinase* (*MERTK*) locus protects against the development of fibrosis and inflammation. The protein is a membrane tyrosine kinase receptor that regulates phagocytes and HSC activation [56]. In particular, the rs4374383 variant is associated with a reduction in hepatic protein expression and protection against fibrosis [56].

Finally, insulin resistance may also be directly involved in increases fibrosis [57,58], and variants in insulin signaling pathway genes may alter the risk of develop fibrosis [59].

## 6. Other Common Genetic Determinants of NAFLD

Other common genetic variants contributing to lipid metabolism come from GWAS. The rs58542926 C>T encoding for the E167K variant in *Transmembrane 6 superfamily member 2* (*TM6SF2*) leads to a decreased expression of the gene and a loss-of-function and increase the risk of NAFLD (Table 1). The E167K variant is associated with increased fat accumulation in the liver, due to a reduction in lipid secretion and/or lipidation of very low-density lipoproteins (VLDL) [19,60]. As this genetic variant also reduces circulating lipids, at the same time it protects against the risk of developing cardiovascular disease [61,62,63].

*Membrane bound O-acyltransferase domain-containing 7* (*MBOAT7*) is a novel gene involved in NAFLD pathogenesis that encodes for a membrane-bound enzyme, which incorporates arachidonic acid and other unsaturated fatty acids into phosphatidylinositol and other phospholipids within the so-called remodeling Lands cycle. Specifically, the common rs641738 C>T variant is in linkage with variation at the 3’ untranslated region of *MBOAT7* and has been associated with downregulation of *MBOAT* at the mRNA and protein levels (Table 1). Carriage of this variant is associated with decreased levels of phophatidyl-inositol containing arachidonic acid in hepatocytes and in the circulation [20,21]. The rs641738 *MBOAT7* variant has been recently linked to the risk of NAFLD, inflammation, and fibrosis [20], but also to higher risk of alcoholic cirrhosis. Moreover, it has also been associated with progression of NAFLD to HCC [64].

Variation at the *Glucokinase regulator* (*GCKR*) gene locus has also been identified as a novel common genetic determinant of NAFLD [65,66,67,68]. GCKR is involved in the regulation of de novo lipogenesis by modulating the influx of glucose in hepatocytes. A common missense variant (rs1260326), encoding for the P446L protein, seems to represent the causal variant associated with the hepatic phenotype [22,69,70] (Table 1). GCKR is implicated in the negative feedback loop that regulates the fructose-6-phosphate pathway by inhibiting glucokinase [67]. When the protein activity is impaired by the presence of the missense variant, GCKR does not inhibit glucokinase in response to high levels of fructose-6-phosphate, thereby constitutively activating hepatic glucose uptake and glycolysis with the consequent generation of acetyl-CoA, a rate-limiting substrate for lipogenesis. This process leads to a reduction of circulating glucose and to an increase in insulin sensitivity. At the same time, it leads to enhanced production of malonyl-CoA, which favors hepatic fat accumulation by inhibiting fatty acid translocation to the mitochondria and beta-oxidation.

Vice versa, recent data demonstrated that the rs4841132 variant of *Protein phosphatase 1 regulatory subunit 3B* (*PPP1R3B*) favors glycogen synthesis in response to excessive energy supply, likely protecting against hepatic fat accumulation in at risk individuals through the reduction of lipogenesis [71]. This common variant is also associated with a reduced risk of progressive liver disease in individuals at high risk of NASH [63]. However, the overall impact of this variant on the risk of liver-related clinical events is still controversial [72].

Overall, the evaluation of the impact of variants in *PNPLA3* and other genes implicated in hepatic lipid metabolism indicates that there is a direct and strong correlation between their effects on hepatic fat accumulation and fibrosis, suggesting that irrespective of the mechanism, excessive storage of fat in hepatocellular lipid droplets determines progressive liver disease [10,73].

## 7. Rare Genetic Determinants of NAFLD

Rare mutations that strongly alter the function of proteins, and that are involved in the regulation of lipid metabolism in hepatic cells, are also involved in NAFLD development.

Apolipoprotein B is the key protein involved in the assembly and secretion of VLDL and chylomicrons (Table 1). Rare variants in *Apolipoprotein B* (*APOB*) have been associated with hypo-betalipoproteinemia, characterized by severe hepatic fat accumulation, with frequent progression to advanced liver disease and HCC. The mechanism is related to the compartmentalization of lipids within hepatocytes [23,74]. In these cases, the *APOB* variants can result in impaired synthesis, stability or secretion of ApoB100 that inhibit the capacity of exporting VLDL from hepatocytes [75]. 

Moreover, *APOB* mutations that occur at the first portion of the protein affect the activity of Apo48 intestinal enterocytes, deregulating the absorption of liposoluble vitamins, specifically A, D, and E, that may be involved in NAFLD development. A recent study demonstrated that rare *APOB* variants predicted to alter protein function were enriched in Italian patients with NAFLD-HCC and associated with higher HDL cholesterol lower triglycerides [76]. In contrast, NAFLD is generally characterized by increased levels of ApoB, which correlates with dyslipidemia and the risk of cardiovascular complications [77].

Moreover, a significant enrichment in rare functional variants in the *Telomerase reverse transcriptase* (*TERT*) have been reported in patients who developed NAFLD-HCC, possibly implicating telomere shortening and cell senescence in the process of tissue aging and hepatic carcinogenesis related to NAFLD [78,79].

Finally, mutations of *LIPA* gene cause lysosomal acid lipase (LAL) deficiency, a severe genetic disorder, are associated with accumulation of cholesteryl esters and triglycerides in hepatocytes. LAL deficiency has variable penetrance and can present with a clinical picture similar to NAFLD in young individuals [80].

## 8. Therapeutic Perspectives

As NAFLD is a multifactorial disease and its development and progression are strongly influenced by the lifestyle, these new advancements in the field of NAFLD may offer the first clinical applications of genetics in targeting preventive interventions to individuals at higher risk of disease progression [81]. In a recent prospective study, for example, *PNPLA3* and *TM6SF2* variants genotyping allowed for the improvement of risk stratification, thereby ameliorating the prediction of NAFLD development in obese adolescents, especially in those born with intrauterine growth retardation favoring insulin resistance [82]. Moreover, adiposity significantly amplifies the genetic risk of NAFLD by interacting with the common variants associated with hepatic fat accumulation in *PNPLA3*, *TM6SF2*, and *MBOAT7* [20,60]. These genetic markers also predict the risk of HCC in patients with NAFLD, independently of the presence of severe fibrosis [62].

As NAFLD patients are at increased risk of developing cardiovascular disease, but the genetic risk variants are quite specific for liver-related complications, polygenic genetic risk scores may be used to predict the liver-related complications of dysmetabolism and to personalize clinical management, e.g., by intensifying the hepatological follow-up in those at higher risk of progression. In particular, the identification of mutations that cause inhibition of lipid secretion, such as those in *TM6SF2* and *APOB*, may help to select patients with high risk of liver disease, but strongly protect against cardiovascular complications [83].

On the other hand, genetic variants identify subgroups of individuals with a distinct pathophysiology of the disease, who may show a different responses to treatment. For example, the carriage of the *PNPLA3* I148M variant is associated with a reduced beneficial effect of the exposure to dapaglifozin, an inhibitor of a sodium-glucose cotransporter-2 (SGLT2), against steatosis, while also with increased hepatic fat accumulation and liver damage associated with the administration of long-acting basal insulin [84,85,86]. These data suggest that genetic risk factors may affect the response or the side effect of drugs. In addition, the carriage of the *PNPLA3* I148M variant blunted also the possible protective effect of omega-3 fatty acids supplementation and statin treatment on NAFLD-related liver damage [86].

Finally, mutated proteins may be directly targeted by drugs, preventing NAFLD progression. For example, in experimental models of NAFLD/NASH in mice wild-type or knock-in for the *Pnpla3* I148M variant, *Pnpla3* silencing by means of antisense oligonucleotides (ASO) was associated with reduced liver inflammation and fibrosis, which was more marked in mice homozygous for the 148M/M mutation [87]. This study paves the way to the development of precision medicine in NAFLD by targeting specific genetic variants that cause the disease, instead of trying to counteract the consequences.

## 9. Conclusions

Genetic factors that influence hepatic fat accumulation play a causal role in NAFLD development. Depending on the genetic mutation, different metabolic pathways are altered in NAFLD, affecting hepatic fat accumulation. The *PNPLA3* I148M protein variant is the main common genetic factor, which also causes alterations in the metabolism of retinol, determining fat accumulation within the lipid droplets in hepatocytes. Furthermore, the rs72613567 variant in *HSD17B13* plays a role in the regulation of retinoic acid metabolism, suggesting that retinol may be involved in NAFLD development. As demonstrated by the effects of variants in mitochondrial proteins involved in redox status regulation, hepatic damage is increased by oxidative stress generated at the level of mitochondria. Finally, in the near future, genetics may have clinical applications by improving liver disease risk stratification in NAFLD patients. Moreover, it may provide the possibility of developing personalized targeted treatments.

## Figures and Tables

**Table 1:** The table shows the main genetic variants involved in nonalcoholic fatty liver disease (NAFLD) with their relative effect sizes, directions, impacts on steatosis, nonalcoholic steatohepatitis (NASH), fibrosis and hepatocellular carcinoma (HCC), mortality in NAFLD, and minor allele frequency in Italy [16,17,18,19,20,21,22,23].

Gene	Variant	Effect Size	Direction	Steatosis	NASH	Fibrosis	HCC	Mortality in NAFLD	Minor Allele Frequency in Italy
*PNPLA3*	I148M	+++	←↑	+	+	+	+	+	0.27
*TM6SF2*	E167K	+++	←↑	+	+	+	+		0.06
*GCKR*	P446L	+	←↑	+					0.30
*MBOAT7*	rs641738	+	←↑	+		+	+		0.44
*HSD17B13*	rs72613567	++	↓		+	+	+		0.22
*IL28B (IFNL3/4)*	rs12979860	+	↓			+			0.36
*MERTK*	rs4374383	+	↓			+			0.36
*APOB*	several	+++	←↑	+		+	+		<0.01

The symbol “+” indicate the power of the specific gene in the induction of the cited phenomena.
